# High Prevalence of Hypovitaminosis D in Adolescents Attending a Reference Centre for the Treatment of Obesity in Switzerland

**DOI:** 10.3390/children9101527

**Published:** 2022-10-06

**Authors:** Pollyanna Patriota, Sylvie Borloz, Inge Ruiz, Thérèse Bouthors, Serge Rezzi, Pedro Marques-Vidal, Michael Hauschild

**Affiliations:** 1 Swiss Nutrition and Health Foundation, 1066 Epalinges, Switzerland; 2 Paediatric Endocrinology and Diabetology Unit, Service of Pediatrics, Department Women-Mother-Child—CHUV, Children’s Hospital—CHUV, Chem. de Montétan 16, 1004 Lausanne, Switzerland; 3 Department of Medicine, Internal Medicine, Lausanne University Hospital and University of Lausanne, 1015 Lausanne, Switzerland

**Keywords:** adolescents, obesity, hypovitaminosis D, Switzerland

## Abstract

Background: Hypovitaminosis D is common in populations with obesity. This study aimed at assessing (1) the prevalence of hypovitaminosis D and (2) the associations between vitamin D levels and cardiovascular risk factors in adolescents attending a reference centre for the treatment of obesity. Design: Cross-sectional pilot study conducted in the paediatric obesity unit of the Lausanne university hospital, Switzerland. Methods: Participants were considered eligible if they (1) were aged between 10 to 16.9 years and (2) consulted between 2017 and 2021. Participants were excluded if (1) they lacked vitamin D measurements or (2) the vitamin D measurement was performed one month after the base anthropometric assessment. Hypovitaminosis D was considered if the vitamin D level was <30 ng/mL (<75 nmol/L). Severe obesity was defined as a BMI z-score > 3 SD. Results: We included 52 adolescents (31% girls, mean age 13 ± 2 years, 33% with severe obesity). The prevalence of hypovitaminosis D was 87.5% in girls and 88.9% in boys. The vitamin D levels were inversely associated with BMI, Spearman r and 95% CI: −0.286 (−0.555; −0.017), *p* = 0.037; they were not associated with the BMI z-score: −0.052 (−0.327; 0.224), *p* = 0.713. The vitamin D levels were negatively associated with the parathormone levels (−0.353 (−0.667; −0.039), *p* = 0.028) and positively associated with the calcium levels (0.385 (0.061; 0.708), *p* = 0.020), while no association was found between vitamin D levels and blood pressure and lipid or glucose levels. Conclusion: almost 9 out of 10 adolescents with obesity in our cohort presented with hypovitaminosis D. Hypovitaminosis D does not seem to be associated with a higher cardiovascular risk profile in this group.

## 1. Introduction

Hypovitaminosis D (i.e., insufficiency and deficiency of vitamin D) is common in children and adolescents with obesity [1], with prevalence rates varying between 72% (United States) and 92–96% (Germany and Russian Federation) [2]. In Switzerland, a recent study on migrant populations showed that the prevalence of hypovitaminosis D was highest in children from Eastern Mediterranean (80%) and African regions (75%) and that the prevalence of severe deficiency was highest in children from Southeast Asian (39%) and Eastern Mediterranean regions (33%) [3]. Although the mechanisms associating obesity and hypovitaminosis D are poorly understood, the main hypothesis is the sequestration of vitamin D by the adipose tissue of obese people [1], thus reducing its bioavailability [4].

The main role of vitamin D is the maintenance of calcium and phosphorus homeostasis. However, vitamin D receptors are found in most human cells and tissues, indicating many extra-skeletal effects. Several studies have shown that vitamin D deficiency is associated with markers of cardiovascular disease (CVD) such as insulin resistance [5] and type 2 diabetes [6], hypertension [7] and dyslipidaemia [8]. For instance, vitamin D levels have been shown to be positively related to serum high-density lipoprotein-cholesterol (HDL-C) and inversely related to total and low-density lipoprotein-cholesterol (LDL-C) and triglycerides. Still, the studies assessing the associations between vitamin D levels and CVD risk factors are scarce, and validation is needed [8].

Hence, this pilot study aimed at assessing (1) the prevalence of hypovitaminosis D and (2) the associations between vitamin D levels and cardiovascular risk factors in adolescents attending a reference centre for the treatment of obesity.

## 2. Materials and Methods

### 2.1. Participants

This study was conducted at the paediatric obesity unit, Department Women-Mother-Child of the Lausanne university hospital, Switzerland. The unit has both an inpatient and an outpatient facility and admits over 200 new children and adolescents per year. The study was carried out by an interdisciplinary team of professionals trained in childhood obesity, specialized in the follow-up and support of children and adolescents, aged 0 to 18 years, who present with diabetes, hormonal, eating or nutrition disorders. The approach is centred on the patient and his/her family, with a cutting-edge practice based on the latest advances in medical research.

### 2.2. Methods

We conducted a single-centre retrospective observational study. Age at anthropometric assessment, gender and nationality were extracted from the electronic medical records. Nationality was categorized as Swiss/other.

Participants were weighed while wearing light clothing, without shoes and accessories, using a scale (SECA GmbH, Hamburg, Germany) with a precision of 0.1 kg. Height was measured using a wall-mounted stadiometer (Holtain Ltd., Crymych, UK) with an accuracy of 1 mm, with participants in the vertical position, wearing light clothes, without head garments and with undone hairstyle and head positioned in the Frankfurt plane [9]. Body mass index (BMI) was computed; BMI z-scores were computed using the WHO Reference 2007 data [10] and the z-Anthro package for Stata, available at www.who.int/tools/growth-reference-data-for-5to19-years/application-tools. This package performs the same calculations as the WHO AnthroPlus (version 1.0.4, 2009) [11].

Blood pressure was measured using an oscillometric device (OMROM-HEM-Healthcare 7113^®^ Intellisense—OMRON, Dalian Co Ltd., China). The measurement was performed on the right arm, supported at the level of the heart. Two measurements were taken in a quiet room, at 5 min intervals, with the participants previously relaxed and lying. Blood pressure percentiles were computed according to the 2017 guidelines [12].

Biological assays were performed by the central laboratory of the Lausanne university hospital. Vitamin D was assessed by liquid chromatography–mass spectrometry; calcium was assessed by the o-cresolphtalein method; parathormone (PTH) was assessed by an immunoenzymatic, solid-phase assay. Total cholesterol was assessed by the cholesterol oxidase phenol 4-aminoantipyrine peroxidase CHOD-PAP method, HDL-cholesterol was assessed by the CHOD-PAP + PEG + cyclodextrin method, and triglycerides were assessed by the glycerol phosphate oxidase method. Fasting plasma glucose was assessed by the glucose hexokinase assay, and insulin by electrochemiluminescence.

Vitamin D insufficiency was considered for vitamin D levels between 20 and 30 ng/mL (50 and <75 nmol/L). Vitamin D deficiency was defined for vitamin D levels < 20 ng/mL (<50 nmol/L). Hypovitaminosis D was considered if the vitamin D levels were <30 ng/mL (<75 nmol/L) [13].

### 2.3. Inclusion and Exclusion Criteria

Participants were considered as eligible if they were (1) aged between 10.0 and 16.9 years; (2) attended the service between 2017 and 2021. Each eligible adolescent was invited to participate in the study. The objectives of the study were presented to the parents/guardians of the adolescents, and signed statements of informed consent were obtained prior to participation. Acceptance supposed that the individual data contained in the electronic medical record (EMR) would be used in the study. Participants who accepted were excluded if (1) they lacked vitamin D measurements or the vitamin D measurement was performed two months after the anthropometric assessment or (2) they received vitamin D supplementation.

### 2.4. Ethical Statement

The study was approved by the Ethics Commission of Canton Vaud (www.cer-vd.ch), reference CER-VD 2021-00537. The full decisions of the CER-VD can be obtained from the authors upon request. The study was performed in agreement with the Helsinki declaration and its former amendments, and in accordance with the applicable Swiss legislation. All participants provided their signed informed consent before entering the study.

### 2.5. Statistical Analysis

Statistical analyses were conducted using Stata version 16.1 (Stata corp, College Station, TX, USA). Descriptive results are expressed as number of participants (percentage) for categorical data or as average ± standard deviation or median [interquartile range] for continuous data. Bivariate between-group comparisons were performed using chi-square or Fisher’s exact test for categorical data and Student’s t-test or Kruskal–Wallis test for continuous data. The associations between serum vitamin D levels and adiposity markers were assessed by Spearman rank correlation, and 95% confidence intervals (95% CI) were obtained by bootstrap. Multivariate analysis of the factors associated with hypovitaminosis D was performed using logistic regression, and the results are expressed as odds ratio (OR) and 95% CI. Statistical significance was assessed for a two-sided test with *p* < 0.05.

## 3. Results

### 3.1. Characteristics of the Participants

**Figure 1:**
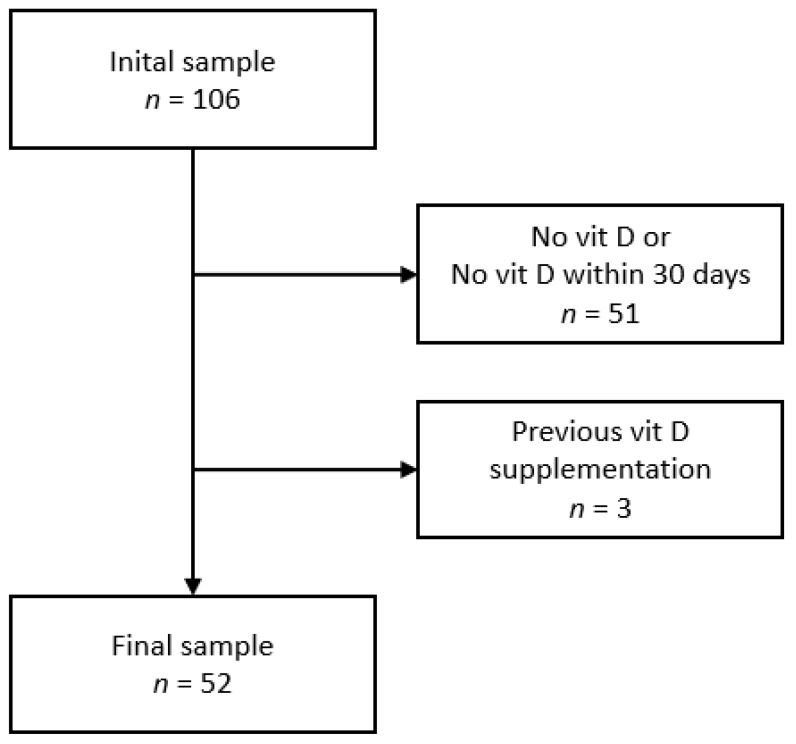
Selection procedure.

Of the initial 106 participants who accepted that their data be analysed, 52 were included. The reasons for exclusion are indicated in Figure 1, and the characteristics of the included and excluded participants are summarized in Supplementary Table S1. Excluded participants were more frequently girls and had a lower BMI z-score.

The characteristics of the participants according to gender are provided in Table 1. Girls were more frequently of Swiss nationality and had higher weight and BMI, while no difference was found for the BMI z-score. For 10 participants, the vitamin D levels were assessed in spring, for 12 in summer, for 15 in autumn and for 15 in winter. The prevalence of hypovitaminosis D was higher in winter and lower in summer, Fisher’s exact test = 0.049 (Supplementary Figure S1).

### 3.2. Prevalence of Hypovitaminosis D

The prevalence of hypovitaminosis D was 89% (33% insufficiency, 56% deficiency), and no differences were found between genders (89% and 88% for boys and girls, respectively, Fisher’s exact test *p* = 1.000) or between nationalities (91% and 86% for Swiss and non-Swiss, respectively, Fisher’s exact test *p* = 0.682). The characteristics of the participants according to presence/absence of hypovitaminosis D are presented in Supplementary Table S2. Besides differences in height, no differences were found for the other clinical markers. Multivariate analysis assessing the factors associated with hypovitaminosis D found no significant associations (Supplementary Table S3).

### 3.3. Associations between Vitamin D Levels, Obesity Markers and Cardiovascular Risk Factors

The bivariate associations between vitamin D levels, obesity markers and cardiovascular risk factors are provided in Table 2. The vitamin D levels were inversely associated with BMI (Spearman r = −0.286, *p* = 0.037) but not with BMI z-scores (Spearman r = −0.052, *p* = 0.713). The vitamin D levels were also negatively associated with age (Spearman r = −0.290, *p* = 0.027) and the PTH levels (Spearman r = −0.353, *p* = 0.032) and positively associated with the calcium levels (Spearman R = 0.385, *p* = 0.018), while no association was found for the other cardiovascular risk factors.

No difference was found between children with obesity and those with severe obesity regarding the vitamin D levels: median and [interquartile range], 19.2 [17.0–25.3] and 17.5 [10.2–24.7] for overweight children and children with obesity, respectively, Kruskal–Wallis test *p* = 0.503.

## 4. Discussion

To the best of our knowledge, this is the first study in Switzerland to verify vitamin D levels and its associations with anthropometric and cardiovascular risk factors in adolescents with obesity.

In our study cohort, we found a high prevalence of hypovitaminosis D of 89%. This value is considerably higher than those previously published for Swiss adolescents (17%) [14,15] but was expected due to the fact that our study focused exclusively on adolescents with obesity, as it has been shown that obesity is a risk factor for hypovitaminosis D [1]. A study carried out in Spain found that almost two-thirds (65.3%) of the adolescents surveyed presented with hypovitaminosis D, this value increasing to 81.1% among children or adolescents with severe obesity [16]. Another Spanish study reported that hypovitaminosis D was present in 68.2% of adolescents with obesity, this value increasing to 81.1% for adolescents with severe obesity [17]. An Italian study found a prevalence of hypovitaminosis D of 91.3% among children and adolescents with obesity [18]. In a Danish study, the prevalence of hypovitaminosis D was 59.4% for children and adolescents with obesity [19]. It is noteworthy that, despite the abundant sunshine, there is a high prevalence of hypovitaminosis D in Southern Europe and Eastern Mediterranean regions [20]. However, it has been shown that in Switzerland, sun’s exposure in wintertime is insufficient for the conversion of vitamin D by the skin [21]. Besides the low sunlight exposure, the most commonly accepted hypothesis for the high prevalence of hypovitaminosis D among adolescents with obesity is the volumetric dilution of vitamin D in fat stores and its decreased bioavailability due to the sequestration of the vitamin by the adipose tissue [22]. However, most recent studies have questioned this hypothesis, as no improvements in vitamin D levels were found after fat loss [23]. The role of vitamin D in the modulation of the adipose tissue and consequent weight gain has been investigated, and preclinical studies have suggested that vitamin D would have effects on adipogenesis, energy homeostasis and the inflammatory response in adipocytes [24]. The hypothesis that people with obesity have a higher prevalence of vitamin D than normal-weight individuals has been confirmed in several studies, also in countries with a high amount of sunlight [1]. New evidence suggests that enzymes involved in vitamin D metabolism might be differently expressed in fat stores of lean and obese individuals; for a review, see [24]. However, the evidence is inconclusive, and further studies are needed. Some studies have also hypothesized that vitamin D insufficiency itself could favour weight gain [25]. Other studies reported that weight loss through lifestyle interventions led to an increase in vitamin D levels [26]. Overall, the prevalence of hypovitaminosis D in our group of children with obesity is in line with the findings of other studies and underlines the need to measure and correct hypovitaminosis D in adolescents with obesity.

### 4.1. Associations between Vitamin D Levels and Cardiovascular Risk Factors

As expected, vitamin D was negatively associated with the PTH levels and positively associated with the calcium levels [22]. The relationship between vitamin D and PTH levels in obesity is complex. Some studies found that the PTH levels are positively associated with the BMI z-score [27,28]. A study conducted in adolescent girls with obesity showed that the ratio of PTH to vitamin D was negatively associated with measures of glucose homeostasis and positively associated with inflammatory markers [29]. Importantly, some associations were lost after adjusting for visceral adipose tissue, suggesting a role of the latter in the association between PTH, vitamin D and insulin resistance in people with obesity. It is hypothesized that hypovitaminosis D in obesity would stimulate PTH secretion, which in turn would increase the renal hydroxylation of vitamin D. This would promote calcium influx into adipocytes, thus promoting lipogenesis and suppressing lipolysis and exacerbating fat deposition [30,31].

We found no association between vitamin D levels and TC, LDL-C, HDL-C, triglycerides, fasting glucose and insulin levels. This result corroborates previous studies carried out in Switzerland [14], Greenland [32] and the USA [5]. However, the US study found an increased chance of having abnormal HDL–cholesterol and impaired insulin resistance among children with hypovitaminosis D. Conversely, our findings do not replicate a previous meta-analysis, where higher serum vitamin D levels were related to a more favourable lipid profile in the paediatric age group [8].

No association was found between vitamin D levels and blood pressure levels. Our results do not replicate a Polish study conducted in 30 adolescents with obesity, where hypovitaminosis D was associated with a higher prevalence of arterial hypertension [7]. Previous studies suggested that vitamin D regulates blood pressure by acting on endothelial cells and smooth muscle cells [33]. Other mechanisms have been proposed, namely, the activation of the renin–angiotensin–aldosterone system, abnormal nitric oxide regulation, oxidative stress, or altered inflammatory pathways [34]. Overall, our results indicate that reduced vitamin D levels were not associated with blood pressure levels in our sample of adolescents with obesity.

### 4.2. Study Limitations

Several limitations should be acknowledged. First, the small sample size and the large number of excluded patients precluded the identification of possibly relevant associations between vitamin D levels and cardiovascular risk factors and might have led to a bias in the interpretation of the results. Despite this study size being comparable to that of others [7], it would be important to increase the sample size to increase the statistical power. Second, the cross-sectional design of the study did not allow establishing causality; the follow-up of the adolescents included will solve this issue. As some information was gathered during the COVID-19 pandemic, it is possible that changes in the participants’ dietary intake might have occurred. Several European surveys including children and adolescents reported a low vitamin D intake [2,3]. A study conducted in 2017 with Swiss adolescents concluded that the “vitamin D intake was below the recommendations” [4]. Hence, it is possible that the changes in vitamin D dietary intake also had a minor effect in our sample of children and adolescents. Finally, it was not possible to consider the impact of some determinants of vitamin D status, such as sun exposure and the use of sunscreens.

## 5. Implications for Practice

In a reference centre for the treatment of adolescent obesity in Switzerland, we found a high prevalence of hypovitaminosis D. The prevalence of hypovitaminosis D was higher in winter and lower in summer, and no differences were found between genders or nationalities.

Our results indicate that screening for vitamin D in adolescents with obesity is imperative. Further studies are needed to establish an adequate vitamin D supplementation for this population, considering formulations, optimization of treatment adherence, and whether vitamin D dosing should be adapted to adolescents’ weight, as it is the case for adults [35].

## 6. Conclusions

We conclude that, in this study, almost 9 out of 10 adolescents with obesity presented with hypovitaminosis D. Hypovitaminosis D did not seem to be associated with higher cardiovascular risk factor levels.

## Figures and Tables

**Table 1:** Characteristics of the participants according to gender.

	Girls	Boys	*p*-Value
Sample size	16	36	
Age (years)	14.0 ± 2.0	13.1 ± 2.2	0.170
Swiss (%)	12 (75.0)	11 (30.6)	0.006
Height (cm)	161 ± 10	160 ± 12	0.868
Height (z-score)	0.6 ± 1.3	0.7 ± 1.3	0.800
Weight (kg)	87.3 ± 18.4	74.8 ± 16.4	0.018
Body mass index (kg/m^2^)	33.4 ± 5.1	28.8 ± 3.7	<0.001
Body mass index z-score	2.9 ± 0.5	2.6 ± 0.5	0.131
Body mass index categories (%)			0.111
Obesity	8 (50.0)	27 (75.0)	
Severe obesity *	8 (50.0)	9 (25.0)	
Vitamin D level (ng/mL)	16.9 [9.7–26.3]	19.2 [12.3–24.0]	0.620 §
Hypovitaminosis D (%)	14 (87.5)	32 (88.9)	1.000

* Defined as a BMI z-score > 3 SD. BMI, body mass index. Results are expressed as number of participants (percentage) for categorical variables and as mean ± standard deviation or median [interquartile range] for continuous variables. Statistical analysis was conducted using the Fisher’s exact test for categorical variables and the Student’s *t*-test or Kruskal–Wallis test (§) for continuous variables.

**Table 2:** Association between vitamin D levels and clinical and biochemical parameters.

	Sample Size	Correlation	*p*-Value
Age (years)	52	−0.290 (−0.556; −0.017)	0.029
Body mass index (kg/m^2^)	52	−0.286 (−0.572; −0.001)	0.037
Body mass index z-score	52	−0.052 (−0.326; 0.223)	0.713
Parathormone	35	−0.353 (−0.675; −0.030)	0.032
Calcium (mmol/L)	32	0.385 (0.070; 0.699)	0.018
Total cholesterol (mmol/L)	49	0.104 (−0.186; 0.395)	0.490
LDL cholesterol (mmol/L)	49	−0.068 (−0.361; 0.224)	0.651
HDL cholesterol (mmol/L)	49	0.224 (−0.051; 0.500)	0.092
Triglycerides (mmol/L)	49	0.233 (−0.059; 0.525)	0.128
Fasting Glucose (mmol/L)	30	0.129 (−0.290; 0.548)	0.546
Insulin	10	−0.036 (−0.849; 0.776)	0.931
Systolic blood pressure (percentile)	31	−0.190 (−0.589; 0.209)	0.351
Diastolic blood pressure (percentile)	31	−0.297 (−0.692; 0.097)	0.139

The results are expressed as Spearman nonparametric correlation coefficient and 95% confidence interval obtained via 1000 bootstraps.

## Data Availability

The participants and their legal representatives did not consent for online posting of their data. No data are available.

## Supplementary Materials

The following supporting information can be downloaded at: https://www.mdpi.com/article/10.3390/children9101527/s1, Figure S1: Prevalence of hypovitaminosis D according to season; Table S1: Characteristics of included and excluded participants; Table S2: Characteristics of participants according to vitamin D status; Table S3: Results of the multivariate analysis of the factors associated with hypovitaminosis D.
