# Supplementary material for: High Prevalence of Hypovitaminosis D in Adolescents Attending a Reference Centre for the Treatment of Obesity in Switzerland
Source: Children (Basel). 2022 Oct 6;9(10):1527. doi: 10.3390/children9101527 (PMC9601272; doi:10.3390/children9101527)
Supplement: Supplementary file 1 [file children-09-01527-s001.zip › children-1921373-supplementary.pdf]

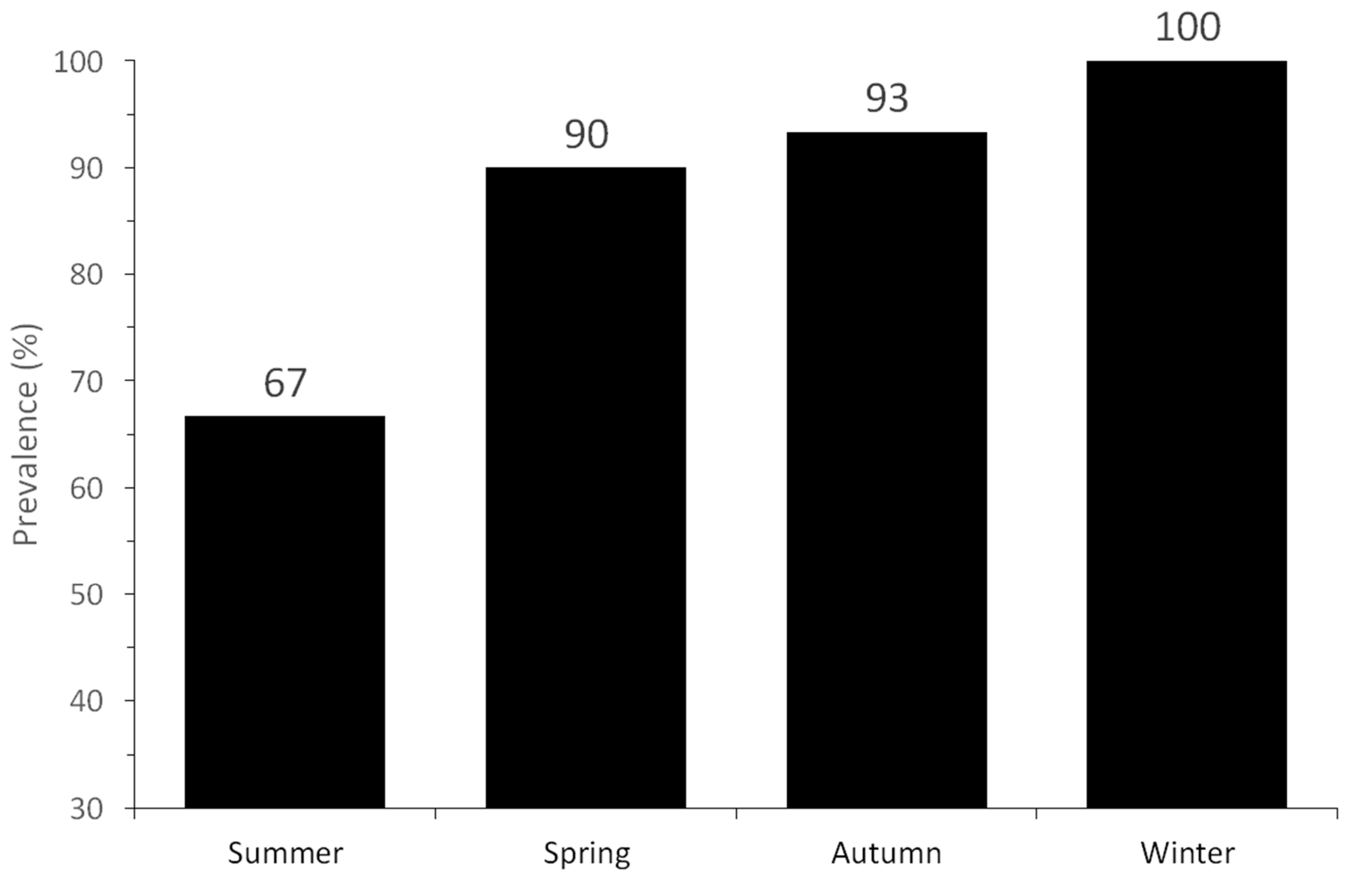

Figure S1. Prevalence of hypovitaminosis D according to season.

Table S1. Characteristics of included and excluded participants.

	Excluded	Included	p-value
Sample size	54	52	
Girls (%)	27 (50.0)	16 (30.8)	0.044
Age (years)	13.2 ± 1.7	13.0 ± 2.1	0.599
Swiss (%)	26 (48.2)	23 (44.2)	0.686
Body mass index (kg/m^2)	29.5 ± 5.8	30.0 ± 4.6	0.653
Body mass index categories (%)			0.055
Obesity	43 (79.6)	32 (61.5)	
Severe obesity	11 (20.4)	20 (38.5)	
Body mass index z-score	2.5 ± 0.6	2.7 ± 0.5	0.041

N=54 for the excluded participants. Results are expressed as number of participants (percentage) or as mean ± standard deviation. Statistical analysis by chi-square for categorical variables and by student's t-test for continuous variables.

Table S2. Characteristics of participants according to vitamin D status.

	Normal	Hypovitaminosis	p-value
Sample size	6	46	
Girls (%)	2 (33.3)	14 (30.4)	1.000
Age (years)	11.9 ± 2.0	13.5 ± 2.1	0.082
Swiss (%)	2 (33.3)	21 (45.7)	0.682
Height (cm)	151 ± 6	162 ± 11	0.019
Weight (kg)	69.8 ± 18.3	79.8 ± 17.7	0.200
Body mass index (kg/m^2)	30.6 ± 6.9	30.2 ± 4.4	0.840
BMI categories (%)			0.379
Obesity	3 (50.0)	32 (69.6)	
Severe obesity	3 (50.0)	14 (30.4)	
Body mass index z-score	3.0 ± 0.4	2.7 ± 0.5	0.114
Initial vitamin D (ng/ml)	34.4 [32.3 - 38.9]	17.2 [10.5 - 22.6]	

Results are expressed as number of participants (percentage) for categorical variables and as mean ± standard deviation or median [interquartile range] for continuous variables. Statistical analysis by Fisher's exact test for categorical variables and by student's t-test for continuous variables.

Table S3. Results of the multivariate analysis of the factors associated with hypovitaminosis D.

	Odds ratio and (95% CI)	p-value
Boys vs. girls	1.63 (0.17 ; 16.0)	0.676
Age (per one-year increase)	1.95 (0.97 ; 3.89)	0.059
Non-Swiss vs. Swiss	0.53 (0.07 ; 4.13)	0.543
BMI (per 1 kg/m^2 increase)	0.87 (0.68 ; 1.10)	0.246

BMI, body mass index; Results are expressed as odds ratio and (95% confidence interval). Statistical analysis by logistic regression. Season could not be included in the model as it lead to a singularity.
